# Prediction of major bleeding events in 1381 patients with essential thrombocythemia

**DOI:** 10.1007/s12185-023-03650-7

**Published:** 2023-09-03

**Authors:** Ruth Stuckey, Jean-Christophe Ianotto, Marco Santoro, Anna Czyż, Manuel M. Perez Encinas, María Teresa Gómez-Casares, Maria Soledad Noya Pereira, Anna Kulikowska de Nałęcz, Aleksandra Gołos, Krzysztof Lewandowski, Łukasz Szukalski, Jesús M. González-Martín, Marta Anna Sobas

**Affiliations:** 1 Hematology Department, Hospital Universitario de Gran Canaria Dr. Negrín, Las Palmas, Spain; 2 Service d’Hématologie et d’Hémostase Clinique, Institut de Cancéro-Hématologie, Centre Hospitalier Universitaire de Brest, Brest, France; 3 Hematology Unit, Department of Health Promotion, Mother and Child Care, Internal Medicine and Medical Specialties (PROMISE), University of Palermo, Palermo, Italy; 4 Department of Hematology and Bone Marrow Transplantation, Wroclaw Medical University, Wrocław, Poland; 5 Hematology Department, Hospital Clínico Universitario de Santiago de Compostela, Santiago de Compostela, Spain; 6 Hematology Department, Hospital Clínico Universitario de A Coruña, A Coruña, Spain; 7 Szpital Wojewódzki, Opole, Poland; 8 Institute of Hematology, Warsaw, Poland; 9 Department of Hematology and Bone Marrow Transplantation, Poznan University of Medical Sciences, Poznan, Poland; 10 Department of Haematology CM UMK in Bydgoszcz, Nicolaus Copernicus University in Toruń, Toruń, Poland; 11 Investigation Unit, Hospital Universitario de Gran Canaria Dr. Negrín, Las Palmas, Spain

**Keywords:** Anticoagulants, Hemorrhage, Mortality, Essential thrombocythemia, Prognosis

## Abstract

The goal of therapy in essential thrombocythemia (ET) is reducing thrombotic risk. No algorithm to predict hemorrhage risk exists. The impact ofanti-platelet, cytoreductive and anticoagulation therapies on risk of major bleeding (MB) was evaluated. MB events were retrospectively analyzed in 1381 ET from 10 European centers. There were 0.286 MB events/person-year. Neither the International Thrombosis Prognostic Score for thrombosis in essential thrombocythemia (IPSET-t) nor the revised IPSET-t (r-IPSET-t) was predictive for hemorrhage-free survival at 10 years (p = 0.092 vs p = 0.1). Ageand leukocyte count were MB risk factors, while low hemoglobin was protective. For ET with extreme thrombocytosis (ExtT) and leukocytosis cytoreduction was not protective. MB were more frequent in ET with ExtT who received anticoagulation. Antiplatelet therapy was not, while anticoagulation was a risk factor for MB (HR 3.05, p = 0.016, CI 1.23–7.56), in particular vitamin K antagonists (22.6% of those treated had a MB event, HR 2.96, p = 0.004, CI 1.41–6.22). Survival at 10 years was associated with hemorrhage (OR 2.54, p < 0.001) but not thrombosis (HR 0.95, p = 0.829). Hemorrhage has a higher risk of mortality than thrombosis. Improved risk stratification for MB is necessary. The choice of anticoagulation, cytoreduction and antiplatelet therapies is an important area of research in ET.

**Supplementary Information:**

The online version contains supplementary material available at 10.1007/s12185-023-03650-7.

## Introduction

Thrombosis and hemorrages are common among patients with the Philadelphia chromosome-negative myeloproliferative neoplasms (MPN), essential thrombocythemia (ET), polycythemia vera and primary myelofibrosis. The main aim for the pharmacological intervention of ET patients is antithrombotic prophylaxis, which may include antiplatelet agents, anticoagulants, and cytoreduction.

ET patients are stratified for thrombotic risk using the International Thrombosis Prognostic Score for thrombosis in Essential Thrombocythemia (IPSET-t) algorithm into a low- (0–1 points), intermediate- (2 points) or high-risk (> 3 points) category, according to age (> 60 years, 1 point), thrombosis history (2 points), cardiovascular risk factors (1 point), and *JAK2*V617F mutation (2 points) [1]. Meanwhile, the revised IPSET-t (r-IPSET-t) algorithm stratifies patients into four groups: very low risk (VLR, no risk factor), low risk (LR, *JAK2* mutation), intermediate risk (IR, ≥ 60 years of age) or high risk of thrombosis (HR, history of thrombosis or ≥ 60 years of age with *JAK2* mutation) [2]. The European LeukemiaNet recommends that ET patients with high thrombotic risk receive low-dose acetylsalicylic acid (LDA) and cytoreduction in the first-line with hydroxyurea (HU), which has a general cytoreductive effect, i.e. lowers both platelet and leukocyte counts, or pegylated interferon-alpha (IFN-α, particularly for younger patients) [3, 4]. In the case of HU resistance, other recommended cytoreductive agents include anagrelide, with selective platelet-lowering effect [5].

We recently showed that the r-IPSET-t score more accurately predicts thrombotic events than the original IPSET-t [6]. However, bleeding risk factors are not well defined and, to date, no dedicated algorithm has been developed to stratify ET patients according to bleeding risk. Moreover, evidence-based guidelines for the prevention of major bleeding (MB) in ET patients are lacking [7].

The aim of this multicenter retrospective cohort study was to study the impact of common anti-thrombotic prophylaxis on bleeding risk and evaluate the impact of bleeding on patient outcome in a real-world setting. The secondary objective was to analyze if the IPSET-t and r-IPSET-t algorithms were useful for predicting risk of hemorrhage for ET patients.

## Methods

### Patients

The medical records of consecutive adult patients diagnosed with ET at various European centers (5 hospitals in Poland, 3 in Spain, 1 in Italy and 1 in France) between 1980 and 2020 were retrospectively evaluated. Patients were included if the ET diagnosis met the corresponding World Health Organization (WHO) at the time of diagnosis.

Driver mutations (*JAK2, CALR* and *MPL*) were determined at diagnosis from 2016 onwards For patients with a diagnosis prior to the routine study of these mutations (*JAK2* and *MPL* mutations were not routinely studied until 2008 onwards, following their inclusion as a major diagnostic criteria by the WHO; *CALR* mutation analysis was added to the 2016 ELN MPN recommendations), the mutation analysis was carried out retrospectively (including for deceased patients), with samples stored at – 80 ºC from diagnosis in biobanks at each collaborating center. Triple negative status was only granted if mutations were negative for *JAK2, CALR* and *MPL.*

The main outcomes of the study were MB event in follow-up, as defined by the ISTH [8]. Death was also recorded as an outcome. Recurrent bleeding event was defined as a second MB event more than 7 days after the first MB event. Follow-up information for all patients, including thrombosis and bleeding events, and disease evolution, was updated in September 2020 through review of medical records.

Information on pharmaceutical treatment received, including antiplatelet, anticoagulation, and cytoreduction (indicated for ExtT; did not include cytapheresis) was also collected. Cytoreductive treatment was defined as the use of HU, anagrelide, or IFN-α. Antiplatelet therapies were defined as the use of LDA or others, and anticoagulation therapies as the use of vitamin K antagonists (VKA), non-vitamin K antagonist oral anticoagulants (NOAC), or low-molecular weight heparin. Patients were treated according to local protocols.

The IPSET-t and r-IPSET-t scores were calculated for each patient with the data from diagnosis [1, 2]. A point was assigned for cardiovascular risk factors if arterial hypertension, diabetes mellitus, and/or active smoking were present in the patient’s medical history.

This study was conducted in accordance with the Declaration of Helsinki. Ethical approval was obtained at each collaborating center. All patient data was anonymized. Individual informed consent was obtained (or waived) according to the local regulations of each center's Institutional Review Board.

### Statistical analysis

Descriptive statistics were used to summarize the characteristics of major hemorrhagic events during follow-up. Data normality was determined according to the Kolmogorov–Smirnov test. Incidence rate was calculated in events/person-year by dividing the total number of bleeding events by the total time of follow-up from diagnosis of all patients. Cox regression was used to predict the dichotomized variables and their time. Hemorrhage-free survival (HFS) was measured from the time of ET diagnosis until the event or date of last follow up. Survival probabilities were estimated using the Kaplan–Meier method and the log-rank test was used for statistical comparison. Missing data were not imputed. P values < 0.05 were considered statistically significant. Analyses were performed using statistical software R Core Team 2020, version 4.1.0 and SPSS, version 22.0.

## Results

A total of 1381 ET patients were included in the cohort, with a median age at diagnosis of 62 years (IQR 48–73; 54.4% ≥ 60 years; patient characteristics are shown in Table 1). With a median follow-up of 66.6 months (IQR 30.0–123.4 months), MB events were registered in 91 patients (6.5%). The incidence rate of bleeding events was 0.286 events/person-year and 50% of events occurred by 5.4 years (IQR 2.3–10.2).

**Table 1:** Characteristics of the cohort of 1381 essential thrombocythemia (ET) patients

Variable	N (%) [Interquartile range]
Male/Female gender	477/904 (34.5%/65.5%)
Age ≥ 60 years at diagnosis	751 (54.4%)
Age at diagnosis (years, median)	62 [48–73]
Blood cell counts at diagnosis:	
Leukocytes × 10^9^/L (median)	9 [8–11]
Hemoglobin g/dL (median)	14 [13–15]
Platelets × 10^9^/L (median)	719 [591–919]
Leukocytes at diagnosis	
≥ 11 × 10^9^/L	378 (29.8%)
Platelets at diagnosis	
≥ 1000 × 10^9^/L	242 (18.8%)
≥ 1500 × 10^9^/L	49 (3.5%)
Mutation	
*JAK2*	898 (65.0%)
*CALR*	264 (19.1%)
*MPL*	24 (2.0%)
Triple negative	176 (12.7%)
Not determined	19
Cardiovascular risk factors at diagnosis	861 (62.4%)
Follow-up time (months, median)	66.6 [30.0–123.4]
Thrombosis after diagnosis	173 (10.8%)
Major bleeding events	91 (6.6%)
Antiplatelet agents	1162 (84.1%)
LDA	1089 (93.7%)
Other	73 (6.3%)
Anticoagulation	85 (6.2%)
Vitamin K antagonist	56 (65.9%)
NOAC	19 (22.4%)
Heparin	8 (9.4%)
Not determined	2 (2.4%)
Cytoreductive therapy	1125 (81.5%)
Hydroxyurea (HU)	947 (84.3%)
Anagrelide	73 (6.5%)
Interferon-α	24 (2.1%)
Other (pharmaceutical)	81 (7.2%)
Exitus	190 (13.8%)

*LDA* low dose acetylsalicylic acid, *NOAC* non-vitamin K antagonist oral anticoagulants

### IPSET-t vs. r-IPSET-t comparison

**Fig. 1:**
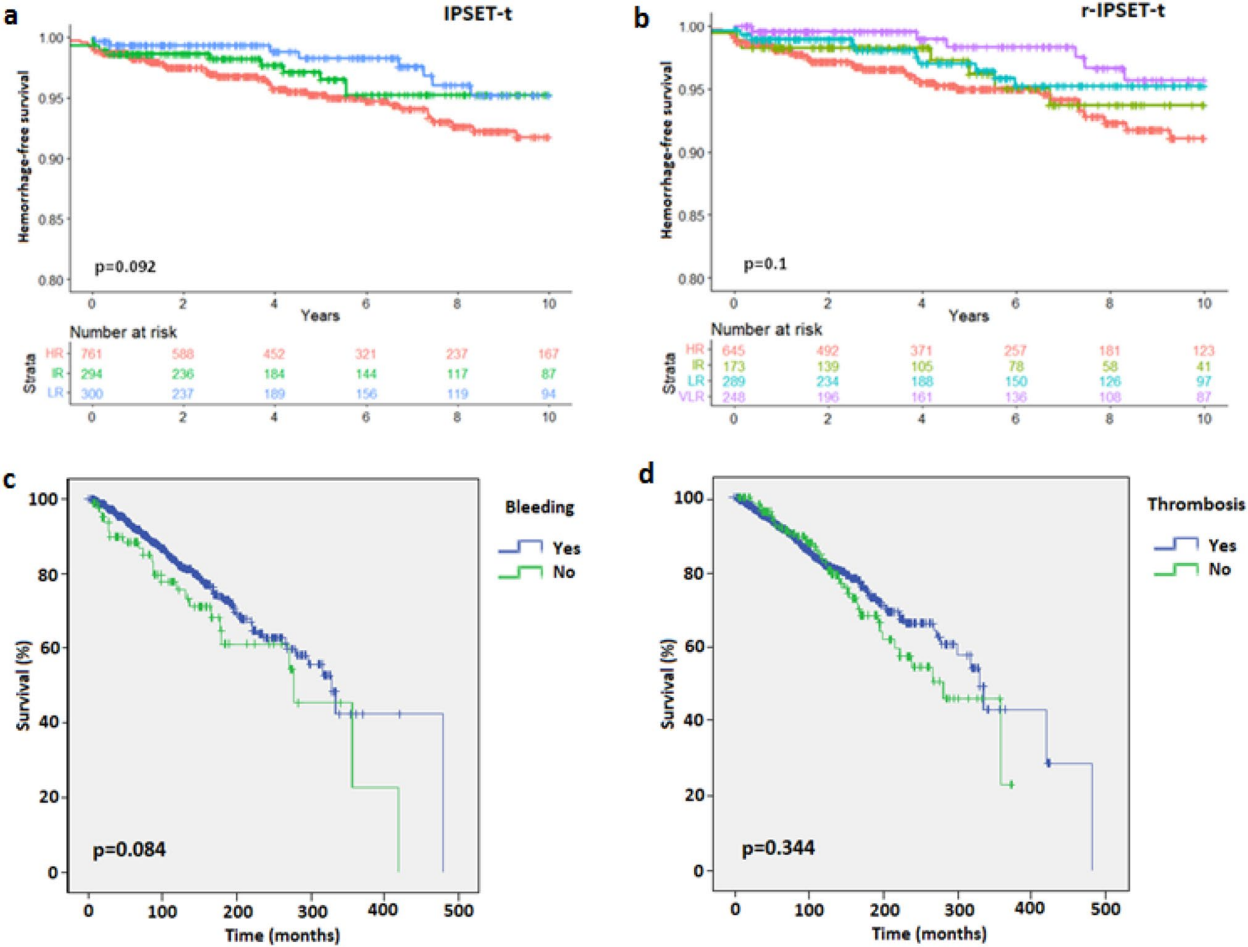
Kaplan–Meier survival curves for hemorrhage-free survival at 10 years comparing the IPSET-thrombosis (IPSET-t, **a**) and revised-IPSET-t (r-IPSET-t, **b**) thrombosis risk groups, and for overall survival comparing patients with major bleeding (**c**) or thrombotic events (**d**). The log-rank test was used for statistical comparison

Most MB events were recorded in the IPSET-t HR group (HR: 61/780, 7.8%; IR: 16/300, 5.3%; LR: 14/301, 4.7%) and the r-IPSET-t HR group (HR: 49/659, 7.4%; IR: 10/177, 5.6%; LR: 20/295, 6.8%; VLR: 12/249, 4.8%). However, there was not a significant difference among the hemorrhage-free survival (HFS) rate at 10 years for either the IPSET-t (p = 0.092) or the r-IPSET-t risk groups (p = 0.1) (Fig. 1a, b). Therefore, we show for the first time that neither the IPSET-t nor the r-IPSET-t score was predictive for HFS.

A comparison was also made of the risk of thrombosis and hemorrhage for each of the r-IPSET-t risk groups. There was a higher risk of thrombotic event vs*.* MB event in the r-IPSET-t HR and IR groups (16.1% vs. 7.4%, p < 0.001, and 13.1% vs. 5.6%, p = 0.003, respectively), but not for the LR and VLR groups (Supplementary Table 1).

### Risk factors for bleeding

With multivariable Cox regression analysis, age at diagnosis and leukocyte count were identified as risk factors for MB, while low hemoglobin (≤ 12 g/dL) at diagnosis was a protective factor (HR 0.78, p = 0.005) (Table 2).

**Table 2:** Univariable and multivariable Cox regression analysis of variables associated with risk of hemorrhage

	Univariable			Multivariable			Multivariable (optimal)		
Variables	HR	*p*-value	95% CI	HR	*p*-value	95% CI	HR	*p*-value	95% CI
Age at diagnosis	1.03	** < 0.001**	1.02–1.05	1.02	0.06	1.0–1.04	1.02	**0.035**	1.0–1.04
Cardiovascular risk factors	1.52	0.11	0.91–2.56	–	–	–	–	–	–
Previous thrombosis	2.12	**0.005**	1.25–3.59	1.35	0.296	0.77–2.36	–	–	–
Leukocyte count at diagnosis	1.1	** < 0.001**	1.05–1.15	1.1	** < 0.001**	1.05–1.15	1.1	** < 0.001**	1.06–1.15
Hemoglobin at diagnosis	0.77	**0.002**	0.69–0.91	0.77	**0.005**	0.65–0.92	0.78	**0.005**	0.65–0.93
Platelet count at diagnosis	1	0.377	1.0–1.0	–	–	–	–	–	–
Mutation type:									
*JAK2* V617F	1.37	0.23	0.82–2.29	–	–	–	–	–	–
*CALR*	0.71	0.27	0.39–1.31	–	–	–	–	–	–
Antiplatelets	0.59	**0.048**	0.35–1.0	1.01	0.978	0.47–2.16	–	–	–
Anticoagulation	4.73	** < 0.001**	2.6–8.61	3.05	**0.016**	1.23–7.56	3.29	** < 0.001**	1.78–6.07
Cytoreduction	4.14	**0.049**	1.01–17.01	2.73	0.175	0.64–11.63	–	–	–

Multivariable analysis was only carried out for variables with statistical significance in the univariable analysis. The proportional hazard assumption (constant HR over time) was confirmed for all variables. Age, leukocyte count, hemoglobin and platelet count at diagnosis were entered as continuous variables

*HR* hazard ratio, *CI* 95% confidence interval

Significant values are shown in bold

No difference in MB risk was observed according to driver mutation, or between patients mutated in *JAK2* or *CALR* who received antiplatelet agents (58/819, 7.1% vs. 14/232, 6.0%, respectively, p = 0.66.

### Treatment impact on hemorrhage

The vast majority of patients with MB received cytoreduction (87/91, 95.6%). Cytoreduction was not a risk factor for MB (Table 2).

Of the 91 patients with a MB event, 81 of them had received antiplatelet agents (90.0%), with most receiving low dose acetylsalicylic acid (LDA, ≤ 100 mg/day). Antiplatelet treatment, and specifically LDA, was not an independent risk factor for MB (Table 2), nor was the frequency of MB events higher for patients who received anagrelide with LDA vs*.* anagrelide alone (5/58, 8.6% vs*.* 3/15, 20.0%).

ExtT (considered as ≥ 1000 platelets × 10^9^/L, n = 242; or ≥ 1500 platelets × 10^9^/L, n = 49) or leukocytosis (leukocytes ≥ 11 × 10^9^/L, n = 349) at diagnosis were not associated with a higher frequency of MB events compared to ET patients with normal levels (≥ 1000 platelets: 7.1% vs*.* 6.0%, p = 0.561; ≥ 1500 platelets: 8.2% vs*.* 6.0%, p = 0.581; ≥ 11 leukocytes: 6.8% vs*.* 6.8%, p = 0.971, respectively). For patients with ExtT and leukocytosis at diagnosis, the protective effect of cytoreductive therapy against MB did not reach significance (≥ 1000 platelets: OR 0.47, p = 0.422; ≥ 1500 platelets: OR 0.74, p = 0.851; ≥ 11 leukocytes: OR 0.88, p = 0.603, respectively). However, a higher frequency of MB events was observed for patients with thrombocytosis ≥ 1000 platelets who received anticoagulation compared to ET patients with thrombocytosis ≥ 1000 platelets who did not receive anticoagulation (23.1% vs*.* 6.1%, OR 4.61, p = 0.03, 95% CI 1.14–18.66). Only 2 patients with thrombocytosis ≥ 1500 platelets received anticoagulation, with no MB reported. No patients with leukocytosis received anticoagulation in this series.

According to multivariable analysis, anticoagulation treatment was a significant risk factor for MB (OR 3.29, p < 0.001; Table 2). A significant difference in haemorrhagic risk was observed for the different types of anticoagulation: VKAs 14/62, 22.6%; NOACs 3/20, 15.0%; heparin 0/8, 0% (p < 0.001). Indeed, VKAs were identified as a significant bleeding risk factor in multivariate Cox regression analysis (HR 2.96, p = 0.004, CI 1.41–6.22; Supplementary Table 2). Of the 62 patients who received VKA and had a history of thrombosis prior to diagnosis (n = 27; 12 arterial and 14 venous events, 1 patient had both), no difference was observed in the frequency of MB events comparing previous arterial (4/12, 33.3%) vs*.* venous thrombosis (6/14, 45.2%; p = 0.619). Of the 90 patients who received anticoagulation, only 6 also received LDA; of these, only 1 patient received VKA and LDA.

### Recurrent bleeding

Six patients had a MB event prior to ET diagnosis (0.4%), 5 patients had a bleeding event < 3 months prior to diagnosis while 1 patient had an event more than 3 years prior to diagnosis.

A total of 16 patients (1.2% of the cohort, 17.6% of those who had a MB event) had a recurrent MB event, with a median time to second event of 5.52 years from diagnosis (all had received cytoreduction). A first MB event was not associated with an increased risk of having a recurrent MB event (bilateral contrast, p < 0.001). From uni- and multivariable analysis, the only independent prognostic variable for a recurrent MB event was age at diagnosis (HR 1.07, p = 0.003, 95% CI 1.02–1.12).

### Survival

There were a total of 190 deaths in our cohort (13.8%). A highly significant association was observed between hemorrhage and death: MB events were responsible for 13.2% of the total deaths (25/190), and 29.1% (25/86) of the MB events resulted in death (OR 2.54, 95% CI 1.55–4.17, p < 0.001). Moreover, when multivariable analysis for survival at 10 years was carried out*,* thrombosis was not a significant risk factor (HR 0.95, p = 0.829, 95% CI 0.59–1.52) while MB was associated with a high risk of mortality (HR 2.25, p = 0.03, 95% CI 1.33–3.79; Supplementary Table 3). An association of marginal significance was also evident in Kaplan–Meier survival curves with MB (p = 0.084, Fig. 1c) but not thrombosis (Fig. 1d, p = 0.344).

## Discussion

The utility of the IPSET-t and r-IPSET-t scores for predicting HFS has not previously been reported. In this cohort of 1381 ET patients, with an incidence density of 0.29 MB events/person-year (similar to earlier reports) [4, 7, 9–11], we show for the first time that neither the IPSET-t nor the r-IPSET-t score was predictive for HFS.

Age and leukocyte count were risk factors for major bleeding and low hemoglobin was protective, in accordance with other published studies [12–14]. Unlike for some other studies, previous haemorrhage was not identified as a prognostic factor for second hemorrhage [10, 11], with only advanced age a risk variable in our cohort.

In relation to treatment effects, and in contrary to a prior analysis of 1104 ET patients, LDA was not an independent risk factor for bleeding complications [10]. Nor did we observe more MB events for LDA with anagrelide vs*.* anagrelide alone, as previously reported [15]. Also in dissent from some reports [16], there was no association between LDA treatment after diagnosis and MB events for *CALR-*mutated ET. Nevertheless, most patients (84%) received LDA and it is possible that the small group of untreated patients had a higher bleeding risk.

ExtT is defined as a platelet value between 1000 and 1500 × 10^9^/L, while platelet count ≥ 1500 × 10^9^/L is considered a risk factor for MB [17] and is an indication to receive cytoreduction [3, 4] as ExtT is frequently associated with acquired von Willebrand syndrome [18]. The frequency of patients with ET with a platelet count of ≥ 1000 × 10^9^/L in our cohort (18.8%) is very similar to the frequency of 22% observed for 3023 patients from the Mayo Clinic with ExtT and a platelet count of ≥ 1000 × 10^9^/L [19]. Yet the prophylactic efficiency of cytoreduction for preventing hemorrhage in patients with ExT (and leukocytosis) was not observed in this cohort (neither ≥ 1000 nor ≥ 1500 × 10^9^/L platelets). However, the majority of patients (81%) and virtually all those with MB (96%) received cytoreduction; therefore, the group of untreated patients is small for comparison. Moreover, we found a significant association between MB and VKA anticoagulation [4, 20], including for patients with high platelet levels. Of the patients who received anticoagulation, less than 7% also received LDA, and only one patient received VKA with LDA. We cannot form conclusions on whether the combination of anticoagulation with LDA is an even greater risk factor for bleeding events, as previously reported, due to the low numbers in our series [21]. Thus, identification of the most appropriate therapy for patients with ExtT, particularly low-risk ET patients—where the risk of thrombosis versus MB is not higher—remains to be determined.

Our study has several limitations due to its retrospective nature. Each center determined if an event constituted MB and may have used different bleeding severity scales and international normalised ratio (INR) optimal ranges during anticoagulation therapy. The location of MB, *CALR* mutation type, and ristocetin cofactor activity were not recorded. In addition, there was no centralized review of diagnosis, thus some ET patients could unknowingly have been cases of prefibrotic myelofibrosis, an entity with a higher bleeding risk than ET [3]. Moreover, many patients received different combinations of antiplatelet and anticoagulation therapies, in addition to cytoreduction in many cases, complicating analysis of the impact of each individual treatment on haemorrhagic risk. The impact of combined therapies on risk of MB may be important. For example, a 2022 study showed that the combination of anticoagulation with LDA increased the risk of MB more than seven-fold compared to LDA alone [22].

The results of this multicenter retrospective study of the largest cohort of ET patients to date reveal that MB at 10 years was a significant risk factor for mortality while thrombosis was not, with nearly 30% of MB events fatal. This observation supports the high mortality rates from MB reported for ET patients in a recent systematic review [7]. Since anticoagulants and antiplatelet agents can increase the bleeding risk in ET, a specific bleeding risk algorithm to help elucidate an individual patient’s bleeding risk would be extremely useful before treatment is recommended. Age, leukocyte counts, and hemoglobin level (all found to be prognostic variables for MB in this study) should be taken into account. Such an algorithm might also include molecular variables, since patients carrying mutations in the genes *ETV6, GATA1, GFI1B*, and *SMAD4,* associated with clonal haematopoiesis of indeterminate potential (CHIP), were found to be at higher risk of hemorrhage [23].

In conclusion, the IPSET-t and r-IPSET-t scores were only developed to predict thrombotic risk and are not predictive for HFS. We show that major bleeding is a more important cause of death than thrombosis, and so improved risk stratification for MB is necessary. The reduction of bleeding risk (and not just the risk of thrombosis) should be included as a therapeutic objective in future clinical trials with ET patients. Also, the choice of antithrombotic therapies (encompassing anticoagulation, cytoreduction and antiplatelets) vs*.* observation in low risk ET patients is an important area of unmet clinical research since MB could be unduly affected by inappropriate therapy.

### Supplementary Information

Below is the link to the electronic supplementary material.

Supplementary file1 (DOCX 19 KB)
