# Supplementary material for: Prediction of major bleeding events in 1381 patients with essential thrombocythemia
Source: Int J Hematol. 2023 Sep 3;118(5):589–95. doi: 10.1007/s12185-023-03650-7 (PMC10615906; doi:10.1007/s12185-023-03650-7)
Supplement: Supplementary file 1 — Supplementary file1 (DOCX 19 KB) [file 12185_2023_3650_MOESM1_ESM.docx]

**SUPPLEMENTARY TABLES**

**Prediction of major bleeding events in 1381 patients with Essential Thrombocythemia**

Ruth Stuckey, Jean-Christophe Ianotto, Marco Santoro, Anna Czyż, Manuel M. Perez Encinas, María Teresa Gómez-Casares, Maria Soledad Noya Pereira, Anna Kulikowska de Nałęcz, Aleksandra Gołos, Krzysztof Lewandowski, Łukasz Szukalski, Jesús M. González-Martín, Marta Anna Sobas

**Corresponding author:** Marta Sobas, Department of Hematology and Bone Marrow Transplantation, Wroclaw Medical University, Poland. [marta.sobas@umed.wroc.pl](mailto:marta.sobas@umed.wroc.pl)

**Supplementary Table 1. Comparative of the frequency of thrombotic and major bleeding events according to the revised IPSET-t (r-IPSET-t) group.**

| r-IPSET-t group | Thrombosis | | | Hemorrhage | | | p-value |
| --- | --- | --- | --- | --- | --- | --- | --- |
|  | **Total** | **Frequency** | **%** | **Total** | **Frequency** | **%** |  |
| HR | 658 | 106 | 16.1 | 659 | 49 | 7.4 | **<0.001** |
| IR | 176 | 23 | 13.1 | 177 | 10 | 5.6 | **0.003** |
| LR | 295 | 29 | 9.8 | 295 | 20 | 6.8 | 0.23 |
| VLR | 249 | 15 | 6 | 249 | 12 | 4.8 | 0.69 |

Significant values are shown in bold. VLR: very low risk, LR: low risk; IR: intermediate risk; HR: high risk.

**Supplementary Table 2. Univariable and multivariable Cox regression analysis of treatment effect on risk of hemorrhage.**

|  | **Variable** | **N** | **Univariable** | | | **Multivariable** | | |
| --- | --- | --- | --- | --- | --- | --- | --- | --- |
|  |  |  | **HR** | **p-value** | **CI** | **HR** | **p-value** | **CI** |
| Cytoreduction | HU | 1281 | 2.50 | **0.004** | 1.310-4.780 | 1.59 | 0.375 | 0.571-4.434 |
|  | Anagrelide | 1281 | 1.03 | 0.996 | 0.4-2.6 | - | - | - |
|  | IFN | 1281 | 1.36 | 0.681 | 0.314-5.877 | - | - | - |
| Anticoagulation | VKA | 1281 | 4.63 | **<0.001** | 2.446-8.755 | 2.96 | **0.004** | 1.409-6.215 |
|  | NOAC | 1281 | 2.56 | 0.125 | 0.737-8.910 | - | - | - |
|  | Heparin | 1281 | 0.99 | 0.377 | 0.986-0.996 | - | - | - |
| Antiplatelets | LDA | 1281 | 0.38 | **<0.001** | 0.41-0.587 | 0.723 | 0.249 | 0.416-1.255 |

HR: hazard ratio; CI: 95% confidence interval; HU: Hydroxyurea; IFN: Interferon-α; LDA: Low dose acetylsalicylic acid; NOAC: Non-Vitamin K antagonist oral anticoagulant; VKA: Vitamin K antagonist.

**Supplementary Table 3. Univariable and multivariable Cox regression analysis of thrombosis and hemorrhage at 10 years as risk factors for death.**

|  | **Univariable** | | **Multivariable** | |
| --- | --- | --- | --- | --- |
| **Variable** | **HR** | **p-value** | **HR** | **p-value** |
| Thrombosis at 10 years | 1.25 | 0.35 | 0.95 | 0.829 |
| Hemorrhage at 10 years | 2.39 | **0.0011** | 2.25 | **0.03** |

HR: hazard ratio
